# Cholinergic muscarinic M_1_/M_4_ receptor networks in dementia with Lewy bodies

**DOI:** 10.1093/braincomms/fcaa098

**Published:** 2020-07-15

**Authors:** Sean J Colloby, Pradeep J Nathan, Ian G McKeith, Geor Bakker, John T O’Brien, John-Paul Taylor

**Affiliations:** f1 Translational and Clinical Research Institute, Faculty of Medical Sciences, Newcastle University, Campus for Ageing and Vitality, Newcastle upon Tyne NE4 5PL, UK; f2 Experimental Medicine, Neuroscience Therapeutic Area, Sosei Heptares, Steinmetz Building, Granta Park, Cambridge CB21 6DG, UK; f3 Department of Psychiatry, University of Cambridge, Cambridge CB2 0QC, UK

**Keywords:** Dementia with Lewy bodies, muscarinic receptors, M1/M4, cholinergic, donepezil

## Abstract

Cholinergic dysfunction is central in dementia with Lewy bodies, possibly contributing to the cognitive and psychiatric phenotypes of this condition. We investigated baseline muscarinic M_1_/M_4_ receptor spatial covariance patterns in dementia with Lewy bodies and their association with changes in cognition and neuropsychiatric symptoms after 12 weeks of treatment with the cholinesterase inhibitor donepezil. Thirty-eight participants (14 cholinesterase inhibitor naive patients, 24 healthy older individuals) underwent ^123^I-iodo-quinuclidinyl-benzilate (M_1_/M_4_ receptor assessment) and ^99m^Tc-exametazime (perfusion) single-photon emission computed tomography scanning. We implemented voxel principal components analysis, producing a series of images representing patterns of inter-correlated voxels across individuals. Linear regression analyses derived specific M_1_/M_4_ and perfusion spatial covariance patterns associated with patients. A discreet M_1_/M_4_ pattern that distinguished patients from controls (W_1,19.7_ = 16.7, *P* = 0.001), showed relative decreased binding in right lateral temporal and insula, as well as relative preserved/increased binding in frontal, precuneus, lingual and cuneal regions, implicating nodes within attention and dorsal visual networks. We then derived from patients an M_1_/M_4_ pattern that correlated with a positive change in mini-mental state examination (*r* = 0.52, *P* = 0.05), showing relative preserved/increased uptake in prefrontal, temporal pole and anterior cingulate, elements of attention-related networks. We also generated from patients an M_1_/M_4_ pattern that correlated with a positive change in neuropsychiatric inventory score (*r* = 0.77, *P* = 0.002), revealing relative preserved/increased uptake within a bilateral temporal-precuneal-striatal system. Although in a small sample and therefore tentative, we posit that optimal response of donepezil on cognitive and neuropsychiatric signs in patients with dementia with Lewy bodies were associated with a maintenance of muscarinic M_1_/M_4_ receptor expression within attentional/executive and ventral visual network hubs, respectively.

## Teaser

We investigated covariance patterns of muscarinic M1/M4 receptors in Lewy body dementia and their correlation with changes in cognition and neuropsychiatric signs after 12 weeks of donepezil treatment. Positive response in cognitive and neuropsychiatric symptoms could be associated with preserved/increased receptors within elements of attention/executive and ventral visual networks, respectively.

## Graphical Abstract

**Figure:**
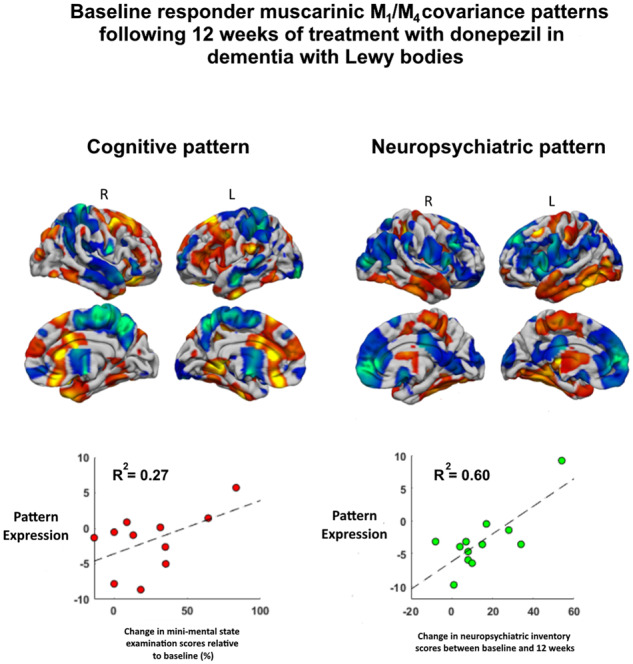

## Introduction

Dementia with Lewy bodies (DLB) is a common form of dementia in older age, where marked cholinergic dysfunction including reduced choline acetyltransferase is a key neurochemical feature and major contributor to the cognitive, sleep and psychiatric symptoms (Aarsland *et al.*, 2003). In Parkinson’s disease dementia which overlaps with DLB in aspects of clinical phenotype, neuropathology and neurochemistry (Outeiro *et al.*, 2019), several dysfunctional cholinergic networks have been proposed to affect the attention, visuoperceptual and memory domains (Gratwicke *et al.*, 2015).

Acetylcholine neurotransmission is facilitated by both muscarinic and nicotinic receptors. Of the former, there are five muscarinic subtypes (M_1_–M_5_), which are widely distributed throughout the central nervous system and brain and are considered to play an important role in learning and memory with M_1_ implicated, in particular, in regulating cognition (Anagnostaras *et al.*, 2003; Erskine *et al.*, 2019). Neuropathological assessment of the various muscarinic subtypes (M_1_-M_4_) has been examined in several brain regions in DLB. Studies have shown decreased M_1_ expression in temporal, hippocampus and parietal areas relative to controls (Ballard *et al.*, 2000; Shiozaki *et al.*, 1999, 2001), and in the striatum compared to Alzheimer’s disease and healthy cases (Piggott *et al.*, 2003). Increased M_1_ in temporal cortex has also been reported (Shiozaki *et al.*, 1999). Elevation of M_2_ in anterior cingulate (Teaktong *et al.*, 2005) and M_3_ in frontal structures (Shiozaki *et al.*, 1999) as well as decreased M_4_ in temporal cortex (Shiozaki *et al.*, 1999) has also been observed in DLB relative to controls. Furthermore, using (R, R) ^125^I-iodo-quinuclidinyl-benzilate (QNB), a muscarinic antagonist whose binding is consistent with the distribution of M_1_/M_4_ receptors found increased binding levels within the insula, cingulate and claustrum in DLB relative to controls (Piggott *et al.*, 2002, 2003).

Understanding how cholinergic receptors are altered at the network level may have implications for treatment. Cholinesterase inhibitors (ChEIs) are the mainstay of symptomatic treatment in DLB (Lam *et al.*, 2009). However, little effort has been made into the impact and optimization of the use of these drugs, for example in relation to target symptoms and dose. At present, there is still a wide range of response heterogeneity to these drugs in DLB with improvements only occurring in about a third of patients in terms of cognition and activities of daily living (Matsunaga *et al.*, 2015), where the picture is even less clear for behavioural and psychiatric symptoms (Matsunaga *et al.*, 2015). While ChEIs show efficacy in improving neuropsychiatric symptoms, it remains to be established if changes in muscarinic receptor networks play a part in neuropsychiatric symptom response to treatment.

One way to examine function at the systems network level is by multivariate approaches such as spatial covariance, a form of principal components (PC) analysis, which overcomes the concept of functional segregation and provides connectivity information between brain regions. In the present study, our aim was to apply spatial covariance to ^123^I-QNB SPECT scans, a ligand with high-binding affinity to M_1_ and M_4_ receptors (Piggott *et al.*, 2002), to investigate muscarinic M_1_/M_4_ connectivity/networks in ChEI naïve patients with DLB. Specifically, we investigated and derived baseline spatial covariance patterns of M_1_/M_4_ that firstly, distinguished DLB from healthy individuals; and secondly in patients, were associated with positive changes in global cognition and neuropsychiatric symptoms after 12 weeks of open-label treatment with the ChEI, donepezil.

## Materials and methods

### Standard protocol approvals, registrations and patient consents

Approval was from the UK Department of Health’s administration of radioactive substances advisory committee (ARSAC) and Newcastle, North Tyneside and Northumberland research ethics committee. Participants gave written informed consent for the study unless they lacked capacity in which case an appropriate consultee (the nearest relative) provided written assent for their participation.

### Participants

We included 38 individuals (14 DLB, 24 elderly controls). DLB participants were recruited from a community-dwelling population of patients referred to old age psychiatry, geriatric or neurology services. Cognitively normal controls were recruited from patient spouses, friends and volunteers. Diagnosis was carried out consensually between two consultant old age psychiatrists using the third report of the DLB consortium 2005 diagnostic criteria (McKeith *et al.*, 2005). Of the 14 patients with DLB, 11 were ‘probable’ and 3 ‘possible’. Autopsy was performed on four cases and received clinico-pathological diagnoses using standardized criteria (Table 1) (Braak *et al.*, 2003; McKeith *et al.*, 2005; Braak *et al.*, 2006). At the time of QNB imaging, DLB patients were naïve to ChEI or anti-Parkinsonian treatment. Participants on any of the following medications were excluded from the study: antipsychotic, cholinergic, anticholinergic and antidepressant medications.

**Table 1:** Participant characteristics

	Controls	DLB				Statistic, *P*-value
*N*	24	14				
Sex (m:f)	15:9	7:7				χ^2^ = 0.6, 0.5
Age	74.1 ± 5.1	74.1 ± 7.1				*F*(1,36) = 0.0003, 0.9
MMSE	28.3 ± 1.5	15.7 ± 6.2				U = 2.0, <0.001
CAMCOG (max 107)	95.0 ± 3.9	54.7 ± 17.5				W_1,13.8_ = 72.0, <0.001
CAMCOG_memory_ (max 27)	22.1 ± 1.9	11.9 ± 5.0				W_1,15.2_ = 53.6, <0.001
CAMCOG_exec_ (max 28)	20.8 ± 4.2	9.1 ± 6.6				*F*(1,36) = 44.6, <0.001
NPI	Na	20.5 ± 19.5				
NPI_hall_	Na	4.2 ± 3.7				
UPDRS III	0.9 ± 1.5	27.4 ± 14.0				*U* = 332.5, <0.001
CAF	Na	4.2 ± 3.3				
Neuropathologically diagnosed DLB cases (*n*=4)						
Case		1	2	3	4	
Braak Tau staging		1	3	3	3	
(1 − 6)						
Braak LB staging		6	6	6	6	
(1 − 6)						
McKeith		3	3	3	3	
(0 − 3)						

Values denote mean ± 1 SD.

MMSE = mini-mental state examination; CAMCOG = Cambridge cognition examination; NPI_hall_ = neuropsychiatric inventory hallucinations subscale; UPDRS = Unified Parkinson’s disease rating scale; CAF = Cognitive fluctuation scale; Na = Not applicable; LB = Lewy body.

Cognitive function was evaluated with the mini-mental state examination (MMSE) and Cambridge Cognitive examination (CAMCOG) (Roth *et al.*, 1986) tests. We also utilized the memory and executive subscales (CAMCOG_memory_, CAMCOG_exec_). Parkinsonism was assessed using part III (motor examination) of the unified Parkinson’s disease rating scale (UPDRS) (Fahn *et al.*, 1987). Neuropsychiatric features and cognitive fluctuations were measured with the neuropsychiatric inventory (NPI) (Cummings *et al.*, 1994) and Clinician Assessment of Fluctuation scale (CAF) (Walker *et al.*, 2000). For analyses (see below), we also considered the NPI hallucinations subscale (NPI_hallucinations_), which was specifically focussed on assessing visual hallucination severity and frequency.

Following their ^123^I-QNB scan, most patients (*n* = 12) were then treated with the ChEI donepezil titrated up to the standard daily clinical dose of 10 mg. After a period of 12 weeks, patients underwent repeated MMSE and NPI assessments.

### Radiochemistry

Employing the technique of Lee *et al.* (1996) (R, R) ^123^I-QNB radiosynthesis was performed, the details of which have been previously described (Pakrasi *et al.*, 2007).

### Acquisition

Individuals were scanned with a triple-head gamma camera (Picker 3000XP), 5 h post-injection of (R, R) ^123^I-QNB using a previously reported imaging protocol (Colloby *et al.*, 2006). Within 4 weeks of the (R, R) ^123^I-QNB scan, participants also underwent ^99m^Tc-exametazime regional cerebral blood flow (rCBF) SPECT scanning (Colloby *et al.*, 2006).

### Spatial pre-processing

Scans were registered to match their specific ^123^I-QNB and ^99m^Tc-exametazime SPECT templates in standard MNI space using the image registration tool FLIRT (https://fsl.fmrib.ox.ac.uk/fsl/fslwiki/FLIRT, accessed 20 July 2020). Information regarding the template images have been reported elsewhere (Pakrasi *et al.*, 2007; Colloby *et al.*, 2008). The registered images were then smoothed with a 10 mm FWHM 3D Gaussian filter.

### Spatial covariance

**Figure 1:**
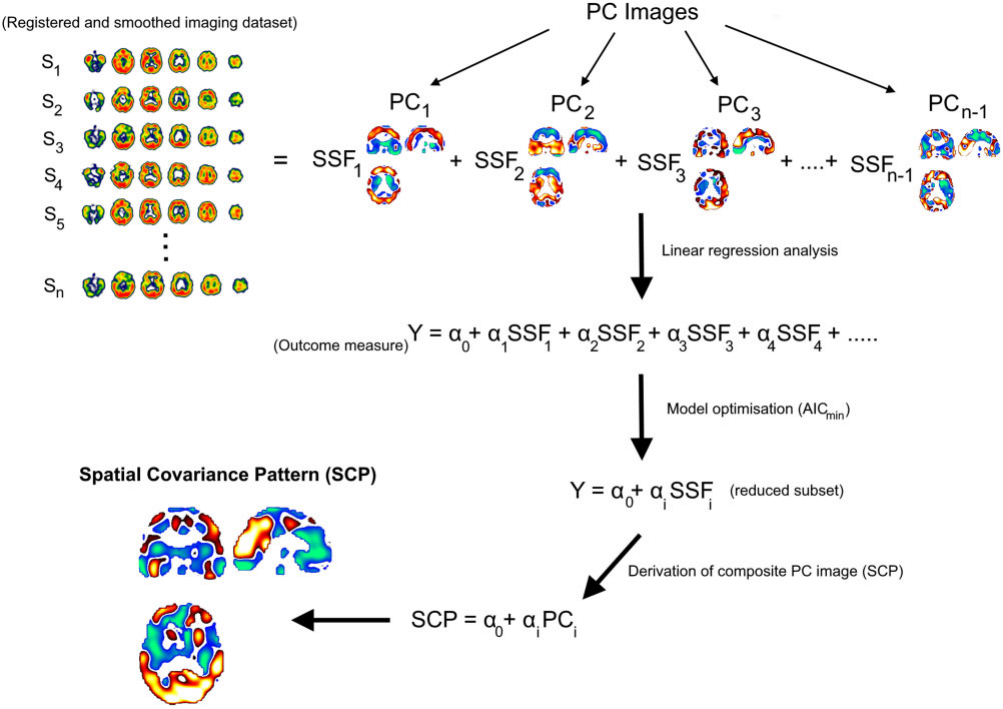
**Analysis pipeline.** Simplified schematic of the spatial covariance analysis pipeline.

Figure 1 depicts a simplified schematic of the spatial covariance analysis pipeline. In detail, spatial covariance analysis was simultaneously applied to ‘*n*’ pre-processed (registered and smoothed) ^123^I-QNB SPECT scans using covariance software (http://www.nitrc.org/projects/gcva_pca/) (Habeck *et al.*, 2005), capturing the major sources of between- and within-group variation, producing (*n* – 1) PC images organized in a descending order of attributed variance. For each PC image, voxels had positive and negative weights representing the sign and strength of voxel covariance that remained fixed across subjects. Specifically, positive and negative voxels were interpreted as concomitant increased and decreased M_1_/M_4_ binding, respectively. The degree to which a subject expressed a PC image (PC_1_, PC_2_,……., PC_*n*__−1_) was by means of the subject-scaling factor (SSF_1_, SSF_2_,…, SSF_*n*__−1_), obtained by multiplying each voxel value of a PC image by the corresponding voxel value in the subject’s QNB scan followed by calculating the total sum of these products to yield a score. Therefore, a high SSF score for a PC image indicates a greater increased binding in voxels with positive weights and a greater decreased binding in voxels with negative weights.

To derive the QNB spatial covariance pattern (SCP_QNB_) that best discriminated DLB from controls; all subject scale factor scores (SSF_1_, SSF_2_,…, SSF_*n*__−1_) were entered into a linear regression model as independent variables with group membership as the dependent measure. Akaike’s information criteria then determined how many SSFs (PCs) should be included in the regression model to achieve optimum trade-off between goodness of fit and model simplicity (Burnham and Anderson, 2002). The set of SSFs (PCs) generating the lowest Akaike’s information criteria value was then chosen as predictor variables for the model that differentiated DLB from controls, where the resulting linear combination and their coefficients formed the composite PC image pattern (SCP_QNB_). The extent to which each subject then expressed the SCP_QNB_ was by the SSF_QNB_, calculated similarly by multiplying each voxel value of the SCP_QNB_ image by the corresponding voxel value in the subject’s QNB scan followed by computing the total sum of these products to produce a score.

Spatial covariance analysis was then conducted on the perfusion ^99m^Tc-exametazime SPECT scans, primarily to assess whether the muscarinic M_1_/M_4_ disease pattern (SCP_QNB_) differed from the perfusion pattern. Therefore, positive and negative weights of these images were viewed as concurrent relative increased and decreased rCBF, respectively. The analysis revealed an SCP_rCBF_ that best separated DLB from controls, with subject expression scores (SSF_rCBF_).

We then derived a ChEI naïve M_1_/M_4_ baseline SCP that correlated with ΔMMSE_rel_b_, describing the percentage change in MMSE between baseline and 12 weeks of post-treatment. Furthermore, we generated ChEI naïve M_1_/M_4_ baseline SCP’s that correlated with ΔNPI_total_ and ΔNPI_hallucinations_, measures that describe changes in global neuropsychiatric and hallucinatory symptoms between baseline and 12 weeks of post-treatment, respectively. This involved performing a separate spatial covariance analysis of the donepezil-treated DLB sample, generating a series of PC images expressed by the SSFs, which in turn were entered into regression models as predictor variables with either ΔMMSE_rel_b_, ΔNPI_total_ or ΔNPI_hallucinations_ as the dependent parameter. The resulting linear combinations with the lowest Akaike’s information criteria values defined the SCP_ΔMMSE_rel_b_, SCP_ΔNPI_total_ and SCP_ΔNPI_hallucinations_, with individual pattern expressions scores of SSF_ΔMMSE_rel_b_, SSF_ΔNPI_total_ and SSF_ΔNPI_hallucinations_, respectively.

Stability and reliability of all SCPs were assessed by bootstrap resampling (1000 iterations), to identify areas that contributed to the patterns with high confidence. This transforms the voxel weights of each SCP into Z maps, computed as the ratio of voxel weight and bootstrap standard deviation. The Z-statistic follows roughly a standard normal distribution where a one-tailed *P* ≤ 0.05 infers a threshold of |Z| ≥ 1.64 (Habeck *et al.*, 2010).

### Statistical analyses

Demographic, cognitive and behavioural variables were tested for normality and variance homogeneity using Shapiro–Wilk and Levene’s tests, respectively. Where applicable, the data were examined using parametric (ANOVA F, Welch’s ANOVA W) and non-parametric (Mann–Whitney *U*, χ^2^) tests, and interpreted as significant if *P* ≤ 0.05. Correlations were examined, where appropriate, with Pearson’s *r* and Spearman’s ρ coefficients. Analysis was conducted using IBM SPSS v.23.0.0.3.

### Data availability

The anonymized data are available to qualified investigators upon request to the corresponding and senior authors.

## Results

### Demographics and clinical characteristics

Table 1 shows demographic and clinical characteristics of study participants. Groups were similarly matched for gender and age, while as expected, cognitive and clinical variables differed (*P* < 0.001).

### Spatial covariance

**Figure 2:**
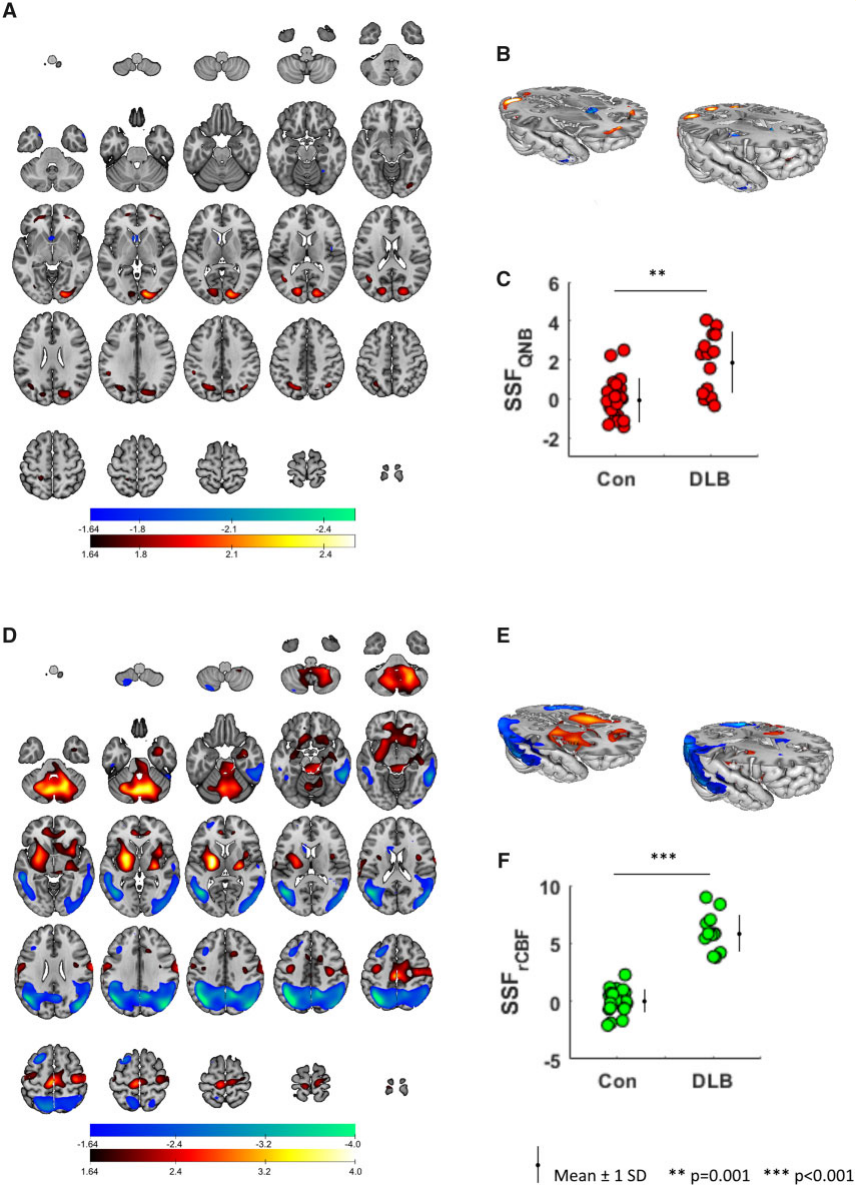
**Spatial covariance patterns in DLB.** Disease M_1_/M_4_ (**A**, **B**) and rCBF (**D**, **E**) baseline covariance patterns in DLB projected onto axial and rendered displays of an MRI brain template. Distribution of subject scores (SSF_QNB_, SSF_rCBF_) across groups, respectively (**C**, **F**). Images displayed neurologically (left is left).

Thirty-seven QNB PC images were initially generated from which PC_1_, PC_3_ and PC_6_ formed the baseline SCP_QNB_ that distinguished DLB from controls (Fig. 2A and B). SSF_QNB_ scores were standardized so that the control mean/SD were 0/1, respectively, and were significantly higher in DLB than controls (mean ± SD; controls = 0 ± 1.0, DLB = 1.9 ± 1.5; W_1,19.7 =_ 16.7, *P* = 0.001, Fig. 2C). The baseline disease pattern was characterized by concomitant bilateral preserved/increased M_1_/M_4_ binding (red regions) in medial/middle frontal gyrus, precuneus, cuneus and lingual gyrus with concomitant decreased binding (blue regions) in right middle/superior temporal gyrus, insula and left caudate regions. Supplementary Table 1 details specific regions contributing to the M_1_/M_4_ disease pattern. Thirty-seven rCBF PC images were then generated from which PC_1,2,3,4_, PC_10_ and PC_11_ derived the associated baseline SCP_rCBF_ (Fig. 2D and E). SSF_rCBF_ scores were similarly standardized and greater in DLB than controls (controls: 0 ± 1.0, DLB: 6.0 ± 1.6; *F*(1,36) = 184.6, *P* < 0.001, Fig. 2F). The rCBF covariance pattern involved bilateral relative increased rCBF (red) in cerebellum, central, lentiform-thalamic, anterior cingulate, insula, amygdala and inferior frontal regions with bilateral relative decreased rCBF (blue) in occipital, temporal and parietal areas. Supplementary Table 2 details specific regions of the rCBF disease pattern.

**Table 2:** Summary data of DLB patients treated with donepezil

	DLB_donepezil_	Statistic, *P*-value
*N*	12	
Sex (m:f)	6:6	
Age	73.5 ± 6.5	
MMSE_b_^a^	18.2 ± 4.9	
MMSE_12w_^a^	22.2 ± 5.3	
MMSE_12w_ – MMSE_b_^a^	4.0 ± 4.0	*W* = 42.5 (*Z* = 2.4), 0.02^b^
ΔMMSE_rel_b_ (%)^a^	25.1 ± 29.0	
NPI_10_b_	20.9 ± 18.5	
NPI_10_12w_	6.1 ± 7.6	
NPI_12w_ – NPI_b_	−14.8 ± 16.8	*W* = 5.0 (*Z* = 2.7), 0.008^b^
NPI_hallucinations__b_	4.0 ± 3.8	
NPI_hallucinations__12w_	1.2 ± 2.5	
NPI_hall12w_ – NPI_hallb_	−2.8 ± 4.1	*W* = 5.0 (*Z* = 2.1), 0.04^b^

Values denote mean ± 1 SD.

_b_ = at Baseline, _12w_ = at 12 weeks.

ΔMMSE_rel_b_ (%) = [(MMSE_12w_ – MMSE_b_)/MMSE_b_] × 100%.

a *n* = 11.

b Related samples Wilcoxon signed-rank test.

Relationship between the disease pattern expressions and age, MMSE, CAMCOG, CAMCOG_memory_, CAMCOG_exec_, total NPI, NPI_hall_, UPDRS III and CAF scores were explored in DLB. Age (*r* = 0.49, *P* = 0.04), CAMCOG (*r* = −0.48, *P* = 0.04) and CAMCOG_memory_ (*r* = −0.52, *P* = 0.03) correlated with the M_1_/M_4_ disease pattern expression, however, after controlling for multiple testing (Bonferroni correction), these associations did not remain significant. Trends between the rCBF pattern (SSF_rCBF_) and these measures did not yield any significant correlations (|*r*| ≤ 0.45, *P* ≥ 0.07).

**Figure 3:**
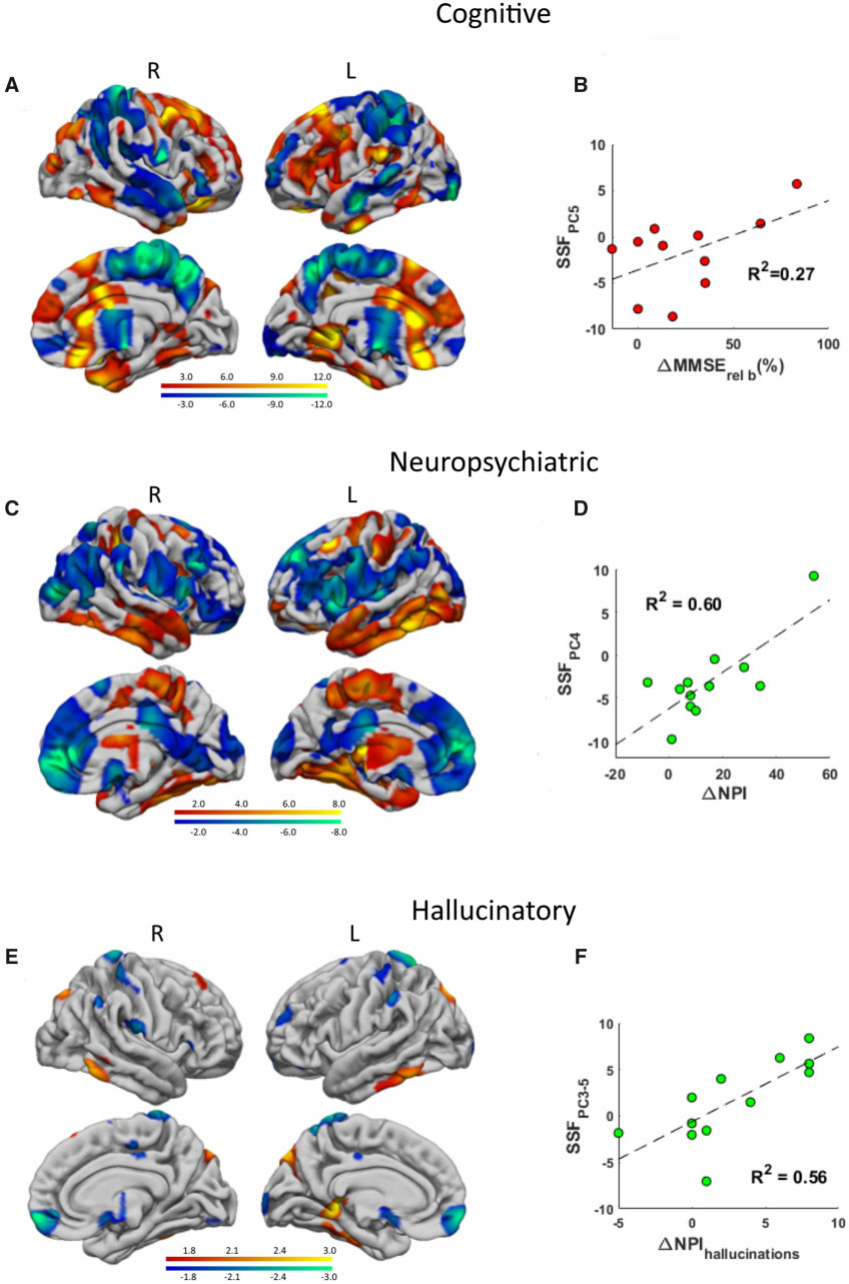
**Responder patterns in DLB.** Baseline cholinergic M_1_/M_4_ cognitive (A), neuropsychiatric (C) and hallucinatory (E) response patterns in DLB.

Data for the donepezil-treated group are presented (Table 2). Eleven QNB PC images were produced from the DLB_donepezil_ sample. Differences in MMSE were found between baseline and 12-week scores (*P* = 0.02). The resultant baseline M_1_/M_4_ pattern (PC_5_) that correlated with ΔMMSE_rel___b_ (*r* = 0.52, *P* = 0.05) was generated and termed the ‘cognitive’ response pattern (Fig. 3A). Figure 3B depicts the pattern expression scores (SSF_PC5_) plotted as a function of ΔMMSE_rel___b_. The covariance pattern consisted of bilateral concurrent preserved/increased M_1_/M_4_ binding in superior/middle/orbitofrontal gyri, inferior temporal, temporal pole, fusiform and anterior cingulate with bilateral concurrent decreased uptake in superior/inferior parietal, precuneus and basal forebrain, superior and middle temporal gyri. Supplementary Table 3 details specific regions contributing to the pattern. Differences in NPI_total_ were also observed between baseline and 12-week post-treatment scores (*P* = 0.008). The M_1_/M_4_ pattern (PC_4_) that was found to be associated with ΔNPI (*r* = 0.77, *P* = 0.002) was derived (‘neuropsychiatric’ response pattern, Fig. 3C). Figure 3D presents the pattern expression scores (SSF_PC4_) plotted as a function of ΔNPI. The covariance pattern comprised of bilateral concomitant preserved/increased M_1_/M_4_ binding in fusiform, parahippocampal gyrus, inferior/middle/superior temporal gyri, precuneus, striatum and superior central regions with bilateral concomitant decreased uptake in medial orbitofrontal, superior/middle frontal gyri, inferior central, insula, cuneus, basal forebrain and posterior cingulate areas (Supplementary Table 4). Lastly, differences in NPI_hallucinations_ were observed between baseline and 12-week post-treatment scores (*P* = 0.04), where the M_1_/M_4_ pattern (PC_3,5_) that correlated with ΔNPI_hallucinations_ was also obtained (*r* = 0.75, *P* = 0.003) (‘hallucinatory’ response pattern, Fig. 3E). Figure 3F shows pattern expression scores (SSF_PC3,5_) plotted as a function of ΔNPI_hallucinations_. The covariance pattern involved bilateral concomitant preserved/increased M_1_/M_4_ binding in fusiform, inferior temporal, precuneus, caudate and left cuneus with bilateral concomitant decreased uptake in anterior and mid-cingulate, medial frontal and superior postcentral gyri (Supplementary Table 5).

## Discussion

This was the first study examining the association between muscarinic M_1_/M_4_ patterns of spatial covariance and improvements in cognitive and neuropsychiatric symptoms with cholinergic therapy in DLB. We undertook a spatial covariance approach of (R, R)^123^I-QNB SPECT scans, an M_1_/M_4_ receptor ligand, in ChEI naïve patients with DLB. This methodology offers connectivity information across brain voxels and therefore functions at the network level, with advantages over traditional univariate techniques where brain voxels are treated as independent. A covariance pattern that discriminated DLB from healthy older individuals was identified and implied M_1_/M_4_ network dysfunction in DLB. We also observed distinct baseline M_1_/M_4_ covariance patterns that were associated with positive changes in MMSE, NPI_total_ and NPI_hallucinations_ following 12 weeks of donepezil treatment. These patterns/networks had clear spatial foci suggesting certain cortical regions and their cholinergic innervation may be selectively relevant for cognitive and neuropsychiatric amelioration through cholinergic therapy.

A muscarinic M_1_/M_4_ covariance pattern was found that distinguished DLB from controls. This disease pattern consisted of concomitant decreased and preserved/increased M_1_/M_4_ binding in several brain regions. The decreased M_1_/M_4_ uptake pattern largely converged on the lateral temporal cortex and insula, where studies have shown their roles in language comprehension and episodic memory (Xie *et al.*, 2012; Goldstein *et al.*, 2017). Thus, the pattern suggests a lateral temporal and insula network of M_1_/M_4_ receptor dysfunction. The disease pattern also showed regions of preserved/increased M_1_/M_4_ activity within frontal, parietal and occipital lobes that implicate two key brain systems important in the symptomatology of DLB, i.e. the dorsal visual stream (Foley *et al.*, 2015) and fronto-parietal (FP) (Markett *et al.*, 2014) networks. This may highlight an M_1_/M_4_ receptor role in both vision and attention in DLB, and chimes with the clinical phenotype of visual hallucinations, visuo-perceptual and attentional deficits (Bradshaw *et al.*, 2006), all of which show response to ChEI (McKeith *et al.*, 2004).

The rCBF disease pattern, that was distinct from the associated M_1_/M_4_ pattern, comprised bilateral relative decreases in occipital, temporal and parietal areas with relative increases in cerebellum, striato-thalamic, limbic, inferior frontal and motor regions in DLB. Regions of relative reduction appear to mainly involve elements of the default mode network (DMN) (precuneus, inferior parietal, lateral temporal cortex) (van den Heuvel and Hulshoff Pol, 2010; Buckner, 2013), where theories infer their contribution to cognitive decline (Seeley *et al.*, 2009). In addition, the relative decreased occipitoparietal perfusion pattern is a characteristic functional deficit that distinguishes DLB from Alzheimer’s disease and healthy individuals (Watson and Colloby, 2016). Our previous fMRI study in DLB showed alterations to FP rather than DMN resting-state networks (Peraza *et al.*, 2015), with FP connectivity strongly linked to cognitive fluctuations (Franciotti *et al.*, 2013; Peraza *et al.*, 2014). Similarly, we also found relative sparing of the DMN in a more recent fMRI study of DLB (Schumacher *et al.*, 2018). However, these studies were in mild disease whereas in the present study, patients were in the moderate/severe stage of illness (mean MMSE: previous 23.6, 22.0; present 15.7). Inconsistency between results could be methodological, or that FP network modulation is perhaps an early feature of this condition, while changes to the DMN emerge later in the course of the disease due to possible increased beta-amyloid and tau co-pathology.

After controlling for multiple testing, no significant correlations were found between the disease pattern expressions (M_1_/M_4_, rCBF) and cognitive/neuropsychiatric variables in DLB. These results were consistent with our previous findings in Parkinson’s disease dementia where we similarly observed no correlations between the disease M_1_/M_4_ pattern expressions with cognitive and neuropsychiatric symptoms (Colloby *et al.*, 2016). The lack of such correlations could be the small sample size, or that covariance disease patterns derived from combined control-patient cohorts characterize a number of composite networks that are less sensitive to specific cognitive and clinical symptoms.

In DLB, we derived an M_1_/M_4_ covariance pattern that positively correlated with improvements in MMSE following 12 weeks of ChEI. This cognitive response pattern showed relative decreases in lateral temporal and parietal regions along with relative preserved/increased binding in prefrontal, temporal pole, fusiform and anterior cingulate. From a network perspective, there were covariant preservation/upregulation in areas converging on key components of the FP (lateral/medial prefrontal), semantic (Tsapkini *et al.*, 2011) (temporal pole), visual (Grill-Spector *et al.*, 2017) (fusiform) and salience (anterior cingulate) networks. Notably, the FP network underpins attention and executive function and these are key cognitive domains which demonstrate improvements in DLB with ChEI treatment (Meng *et al.*, 2019). Therefore, the results may imply that a relative M_1_/M_4_ receptor maintenance within some elements of attention/executive/visual/salience networks are pre-requisite for ChEI treatment response, and could point towards the potential relevance of these networks and their cholinergic innervation as a predictor of positive treatment response to cognition in DLB.

We also identified an M_1_/M_4_ covariance pattern that correlated with a decrease in the total NPI score. This ‘neuropsychiatric’ response pattern showed relative decreases in lateral/medial prefrontal, cuneus, insula, basal forebrain and posterior cingulate regions with relative preserved/increased uptake in lateral/medial temporal, precuneus and striatum. In terms of networks, the covariant preserved/increased uptake appears to involve a bilateral temporal-precuneal-striatal network that might implicate elements/hubs of the ventral visual stream, DMN and frontostriatal circuits. As an adjunct to the neuropsychiatric pattern and that visual hallucinations are one of the core features of DLB, we generated an M_1_/M_4_ covariance pattern that correlated with changes in NPI_hallucinations_, revealing a pattern which was associated with decreased visual signs. This more specific pattern (contained in entirety within the broader, distributed neuropsychiatric pattern described above) showed bilateral preserved/increased binding in fusiform, inferior temporal, cuneus/precuneus and caudate with bilateral decreased uptake in anterior/mid-cingulate, frontal and postcentral areas. The concomitant preserved/increased pattern suggests contributions from primary visual, visual association and DMN regions, i.e. an inferior temporal-occipito-parietal network. Notably, a recent study showed an association between visual hallucinations and decreased metabolic connectivity in the right lateral temporal and bilateral fusiform regions, outlining their potential impact on visual symptoms in DLB (Iaccarino *et al.*, 2018).

The present findings support the conjecture that a number of brain regions are central to the neuropsychiatric profile of DLB, i.e. fusiform, inferior temporal gyrus, precuneus and striatum, and that cholinergic maintenance of these structures is a precondition for ChEI treatment response of neuropsychiatric symptoms, particularly visual hallucinations in DLB. The underlying neuropathology of neuropsychiatric symptoms in DLB and neurodegenerative disorders in general is poorly understood. While ChEIs do improve neuropsychiatric symptoms, they have modest effects on psychotic symptoms including delusions and hallucinations (Armstrong and Weintraub, 2017). Results from the current study suggest changes in M_1_/M_4_ receptor networks may play a role in neuropsychiatric symptom response to treatment. In line with symptoms of hallucinations and delusions occurring in psychotic disorders without dementia, there is increasing evidence that dysregulated M_1_/M_4_-mediated modulation of glutamate and dopamine in frontal-temporal-striatal regions contribute to the development of these symptoms (Erskine *et al.*, 2019). Treatment options beyond ChEIs for neuropsychiatric symptoms are very limited, with antipsychotics being associated with significant risks (Armstrong and Weintraub, 2017). Our findings support ongoing development of M_1_/M_4_ agonist drugs to treat these symptoms in DLB (Erskine *et al.*, 2019).

Finally, we noted significant overlap between regions associated with both cognitive and neuropsychiatric improvement, i.e. inferior temporal gyrus, temporal pole, fusiform and putamen, and aligns with the known multi-symptom benefits of cholinergic treatment in DLB (McKeith *et al.*, 2000). Overall, this observation suggests that these regions, in particular, may have a core role (from an M_1_/M_4_ perspective) in the clinical phenotype of DLB and relevance as important therapeutic targets for designing outcome measures in trials and for clinical assessment in practice.

Some limitations of the study warrant discussion. First, the study had a small sample size and therefore results should be interpreted as tentative. Small samples could also explain the lack of correlations observed with the baseline behavioural and clinical symptoms. Second, given the historical nature of the dataset, MoCA assessments were not widely in use at that time of the study. However, MMSE has shown consistent benefits in ChEI trials whereas MoCA remains unproven (Birks and Harvey, 2018). Third, the SPECT ligand used (^123^I-QNB) is non-selective for muscarinic receptor subtypes (i.e. M_1_ vs. M_4_) and hence there is uncertainty regarding which receptor subtype contributed to the pattern of changes observed in specific areas of these networks. Both receptor subtypes have been shown to be associated with neuropsychiatric symptoms in DLB, albeit with non-selective ligands (Ballard *et al.*, 2000; Teaktong *et al.*, 2005). While a recent *in vivo* PET imaging study using a new M_4_ selective ligand, ^11^C-MK-6884, showed 20–50% reduction in receptor binding in frontal and temporal cortices in patients with moderate to severe Alzheimer’s disease (Wang *et al.*, 2019). This suggests there is reduced acetylcholine release and potentially selective changes in cortical M_4_-mediated signalling in dementia, consistent with similar cortical muscarinic receptor reductions observed in Alzheimer’s disease with the non-selective ligand ^123^I-QNB (Pakrasi *et al.*, 2007). These findings suggest the pattern of cortical and subcortical changes that we observed in DLB may be driven by changes in both M_1_ and M_4_ receptor subtypes. Fourth, given the inclusion of severe DLB cases in this study, we cannot exclude the possibility that some patients will have significant mixed pathology, which in turn could affect the results. Although reassuringly, of the four cases that did have autopsy confirmation of their diagnoses, none appeared to have significant Alzheimer’s disease co-pathology. Finally, the investigation on the treatment-related changes had no placebo comparison group and replication of this study with newer selective M_1_ and M_4_ receptor PET ligands along with neuropsychological assessments, which align more with the cognitive deficit profile of DLB, may provide more specific cholinergic receptor response patterns. The study also had strengths including clinical assessment of DLB patients, muscarinic SPECT scans conducted prior to ChEI treatment and follow-up assessments from the effects of 12 weeks of ChEI treatment. Autopsy confirmation of diagnoses was available for a minority of cases confirming clinical diagnoses, though not for the entire patient sample. It is also worth noting that since responder M_1_/M_4_ covariance patterns correlated with changes in MMSE/NPI scores, and because multiple factors may affect both cognition and neuropsychiatric symptoms at baseline in DLB, the observed changes were assumed specific to cholinergic modification.

In summary, we infer a number of dysfunctional M_1_/M_4_ receptor and perfusion networks associated with DLB. The relevance of these networks may be important in terms of their contribution to clinical features of DLB, in particular, the attentional deficits and hallucinations. The use of ChEIs could improve the symptoms; but there is marked heterogeneity between DLB patients in response to these agents and impractical to reliably predict on clinical grounds who might respond to these drugs. Although speculative and in a small sample, we identified patterns that suggest those with a pre-existing M_1_/M_4_ receptor maintenance of ‘attentional/executive’ and ‘visual’ networks may experience cognitive and neuropsychiatric symptomatic improvement with ChEI treatment, respectively. These findings provide further insights into therapies targeted at improving cholinergic neurotransmission (mediated via muscarinic M_1_ and M_4_ receptor agonists) and treatment outcomes in DLB.

## Funding

Medical Research Council UK [grant number G9817682]. National Institute for Health Research Dementia Biomedical Research Unit at Cambridge University Hospitals National Health Service Foundation Trust and the University of Cambridge. The National Institute for Health Research Newcastle Biomedical Research Centre in Ageing and Chronic Disease and Biomedical Research Unit in Lewy Body Dementia based at Newcastle upon Tyne Hospitals National Health Service Foundation Trust and Newcastle University. Industry grant from Sosei Heptares, Cambridge, UK.

## Competing interests

S.J.C. reports no disclosures. P.J.N. is an employee of Sosei Heptares and holds shares in the company. I.G.M. has been a consultant for GE and Bayer Healthcare. G.B. is an employee of Sosei Heptares. J.T.O’B. has been a consultant for GE Healthcare, Lilly, Bayer Healthcare, TauRx, Axon, Eisai and Roche. J.-P.T. has been a consultant of Kyowa Kirin and Sosei Heptares, received grant funding from Sosei Heptares and honoraria for talks from GE Healthcare.

## Supplementary Material

fcaa098_Supplementary_Data

## Abbreviations

CAMCOG: Cambridge Cognitive examination
ChEI: cholinesterase inhibitor
DLB: dementia with Lewy bodies
DMN: default mode network
FP: fronto-parietal
MMSE: mini-mental state examination
MNI: Montreal neurological institute
NPI: neuropsychiatric inventory
PC: principal components
QNB: iodo-quinuclidinyl-benzilate
rCBF: regional cerebral blood flow
SCP: spatial covariance pattern
SPECT: single-photon emission computed tomography
SSF: subject scale factor
