# Supplementary material for: Cholinergic muscarinic M1/M4 receptor networks in dementia with Lewy bodies
Source: Brain Commun. 2020 Jul 15;2(2):fcaa098. doi: 10.1093/braincomms/fcaa098 (PMC7475694; doi:10.1093/braincomms/fcaa098)
Supplement: fcaa098_Supplementary_Data [file fcaa098_supplementary_data.pdf]

Supplementary Table 1. Location of regions contributing to the cholinergic M_1/M_4 disease pattern in DLB.

Hemisphere	MNI coordinates	Region	Z score
R	23, 53, 1	Medial frontal gyrus	2.0
L	-11, 55, 1	Medial frontal gyrus	1.9
R	42, 48, 3	Middle frontal gyrus	1.8
L	-33, 44, -2	Middle frontal gyrus	1.7
R	40, -65, 40	Precuneus	2.1
L	-28, 65, 40	Precuneus	1.9
R	13, -88, 3	Lingual gyrus	2.2
L	-13, -87, 3	Lingual gyrus	1.8
R	14, -89, 10	Cuneus	2.2
L	-16, -85, 10	Cuneus	1.9
R	59, 6, -35	Middle temporal gyrus	-1.7
R	56, 12, -37	Superior temporal gyrus	-1.8
L	-5, 12, 2	Caudate	-2.1
R	45, -9, 17	Insula	-1.9

Supplementary Table 2. Location of regions contributing to the rCBF disease pattern in DLB.

Hemisphere	MNI coordinates	Region	Z score
R	18, -65, -35	Posterior cerebellum	3.5
L	-12, -64, -35	Posterior cerebellum	3.0
R	34, -7, -21	Amygdala	1.9
L	-26, -4, -18	Amygdala	1.9
R	23, 22, -19	Inferior frontal	1.8
L	-14, 23, -19	Inferior frontal	2.1
R	28, 1, 1	Putamen	1.8
L	-25, -2, 1	Putamen	3.6
R	15, 43, 1	Anterior cingulate	2.3
L	-10, 44, 1	Anterior cingulate	2.5
R	21, -19, 6	Thalamus	2.7
L	-16, -21, 6	Thalamus	2.5
R	37, 12, 6	Insula	2.3
L	-40, -2, 6	Insula	1.9
R	66, -4, 20	Postcentral gyrus	2.0
L	-65, -12, 20	Postcentral gyrus	2.0
R	62, -8, 26	Precentral gyrus	2.2
L	-63, -7, 26	Precentral gyrus	2.1
L	-24, -1, -11	Basal forebrain	2.2
R	60, -48, -16	Inferior temporal gyrus	-3.2
L	-55, -32, -16	Inferior temporal gyrus	-1.9
R	65, -40, -14	Middle temporal gyrus	-2.9
L	-57, -37, -5	Middle temporal gyrus	-3.2
R	39, -92, -5	Inferior occipital gyrus	-2.2
L	-39, -75, -5	Inferior occipital gyrus	-1.9
R	40, -87, 2	Middle occipital gyrus	-2.8
L	-37, -71, 2	Middle occipital gyrus	-2.6
R	60, -43, 24	Inferior parietal	-2.5
L	-52, -44, 29	Inferior parietal	-2.1
R	9, -60, 29	Precuneus	-3.1
L	-1, -64, 24	Precuneus	-2.4
R	44, -57, 53	Superior parietal	-2.5
L	-40, -59, 53	Superior parietal	-2.8
L	-12, 15, 15	Caudate	-2.0
L	-28, 58, 5	Superior frontal gyrus	-1.9

Supplementary Table 3. Regions contributing to the cholinergic M_1/M_4 'cognitive' response pattern in DLB.

Hemisphere	MNI coordinates	Region	Z score
R	7, 25, 54	Superior frontal gyrus	11.3
L	-6, 24, 52	Superior frontal gyrus	9.7
R	33, 43, 3	Middle frontal gyrus	4.1
L	-33, 41, 7	Middle frontal gyrus	4.5
R	6, 29, -19	Orbitofrontal cortex	10.3
L	-2, 33, -20	Orbitofrontal cortex	12.2
R	57, -44, -35	Inferior temporal gyrus	5.8
L	-53, -13, -35	Inferior temporal gyrus	13.5
R	40, 16, -33	Temporal pole	9.2
L	-40, 16, -33	Temporal pole	3.3
R	53, -54, -16	Fusiform	7.3
L	-52, -59, -16	Fusiform	4.2
R	14, 32, 15	Anterior cingulate	5.8
L	-6, 36, 14	Anterior cingulate	4.0
L	-27, 9, 0	Putamen	11.3
R	32, -93, 8	Middle occipital gyrus	7.7
R	34, -43, 65	Superior parietal	-3.2
L	-24, -41, 65	Superior parietal	-3.6
R	41, -40, 60	Inferior parietal	-8.7
L	-33, -41, 60	Inferior parietal	-8.0
R	31, -63, 35	Precuneus	-7.0
L	-22, -67, 35	Precuneus	-6.2
R	52, 9, -19	Superior temporal gyrus	-5.4
L	-51, 22, -8	Superior temporal gyrus	-1.7
R	68, -17, -11	Middle temporal gyrus	-2.3
L	-56, -5, -23	Middle temporal gyrus	-10.7
L	-30, -16, -16	Hippocampus	-3.3
L	-18, -3, 4	Globus pallidus	-2.2
L	-5, -96, -3	Occipital pole	-1.7
L	-16, -1, -13	Basal forebrain	-3.1

Supplementary Table 4. Regions contributing to the cholinergic M_1/M_4 'neuropsychiatric' response pattern in DLB.

Hemisphere	MNI coordinates	Region	Z score
R	57, -33, -29	Fusiform	5.9
L	-49, -28, -29	Fusiform	6.7
R	41, -27, -29	Parahippocampal gyrus	6.4
L	-32, -27, -29	Parahippocampal gyrus	6.8
R	62, -25, -23	Inferior temporal gyrus	6.5
L	-60, -16, -23	Inferior temporal gyrus	6.2
R	61, -14, -16	Middle temporal gyrus	4.0
L	-59, -6, -16	Middle temporal gyrus	4.7
R	56, -4, -8	Superior temporal gyrus	3.3
L	-60, -5, -8	Superior temporal gyrus	8.5
R	16, 17, -1	Caudate	3.7
L	-15, 18, -1	Caudate	2.8
R	28, 8, 9	Putamen	3.7
L	-22, 10, 9	Putamen	6.3
R	18, -40, 57	Precuneus	3.3
L	-11, -40, 57	Precuneus	3.9
R	18, -24, 68	Superior precentral gyrus	4.7
L	-16, -23, 68	Superior precentral gyrus	8.0
L	-30, -11, -18	Hippocampus	2.5
L	-16, 29, -14	Inferior frontal gyrus	7.1
R	8, 49, -19	Medial orbitofrontal gyrus	-7.0
L	-9, 47, -19	Medial orbitofrontal gyrus	-3.2
R	23, 29, 43	Superior frontal gyrus	-5.8
L	-12, 26, 51	Superior frontal gyrus	-7.7
R	32, 30, 39	Middle frontal gyrus	-5.4
L	-25, 30, 35	Middle frontal gyrus	-4.0
R	41, 12, 4	Insula	-7.4
L	-37, 13, 4	Insula	-5.8
R	13, -93, 14	Cuneus	-2.9
L	-8, -92, 14	Cuneus	-3.3
R	64, -12, 20	Inferior postcentral gyrus	-9.1
L	-60, -13, 20	Inferior postcentral gyrus	-3.5
R	20, 5, -13	Basal forebrain	-5.1
L	-19, 5, -13	Basal forebrain	-6.9
R	11, -58, 12	Posterior cingulate	-4.0
L	-8, -58, 12	Posterior cingulate	-3.3

Supplementary Table 5. Regions contributing to the cholinergic M_1/M_4 'hallucinatory' response pattern in DLB.

Hemisphere	MNI coordinates	Region	Z score
R	57, -54, -21	Fusiform	1.9
L	-54, -49, -21	Fusiform	1.8
R	58, -55, -15	Inferior temporal gyrus	2.0
L	-57, -52, -15	Inferior temporal gyrus	1.9
R	16, 20, -3	Caudate	2.2
L	-15, 24, -3	Caudate	2.2
R	28, -76, 48	Precuneus	2.0
L	-20, -76, 48	Precuneus	2.1
L	-20, -76, 16	Cuneus	2.1
R	8, 48, -8	Anterior cingulate	-1.9
L	-4, 48, -8	Anterior cingulate	-1.8
R	8, 60, -8	Medial frontal gyrus	-2.0
L	-4, 60, -8	Medial frontal gyrus	-2.0
R	7, -21, 42	Mid cingulate gyrus	-1.9
L	-2, -24, 42	Mid cingulate gyrus	-1.7
R	16, -36, 72	Superior postcentral gyrus	-1.9
L	-12, -40, 72	Superior postcentral gyrus	-1.9
